# Supplementary material for: Development and initial validation of the German version of the Exergame Enjoyment Questionnaire (EEQ-G)
Source: PLoS One. 2023 Jun 8;18(6):e0286556. doi: 10.1371/journal.pone.0286556 (PMC10249825; doi:10.1371/journal.pone.0286556)
Supplement: S1 File — (PDF) [file pone.0286556.s001.pdf]

Supplementary Files
-
Development and Initial Validation of the German Version of the Exergame Enjoyment Questionnaire (EEQ-G)

Patrick Manser [1], Simone Huber [1, 2], Julia Seinsche [1], Eling D. de Bruin [1, 3, 4], Eleftheria Giannouli [1, 5]

(1) Motor Control and Learning Group – Institute of Human Movement Sciences and Sport, Department of Health Sciences and Technology, ETH Zurich, Zurich, Switzerland;
(2) Physiotherapy and Occupational Therapy Research Centre, Directorate of Research and Education, University Hospital Zurich, Zurich, Switzerland;
(3) Division of Physiotherapy, Department of Neurobiology, Care Sciences and Society, Karolinska Institute, Stockholm, Sweden;
(4) Department of Health, OST – Eastern Swiss University of Applied Sciences, St. Gallen, Switzerland;
(5) Department of Sport, Exercise and Health, Division of Sports and Exercise Medicine, University of Basel, Basel, Switzerland

Corresponding Author:

Patrick Manser

E-Mail: patrick.manser@hest.ethz.ch

Overview:

This file contains the EEQ-G (pages 2 – 3). The original EEQ (in English) is available on pages 4 – 5 and in its original publication:

> *Alexander Fitzgerald, Sam Huang, Kyle Sposato, Dongjie Wang, Mark Claypool, and Emmanuel Agu. "The Exergame Enjoyment Questionnaire (EEQ): An Instrument for Measuring Exergame Enjoyment", In Proceedings of the Hawaii International Conference on System Sciences (HICSS), Maui, Hawaii, January 7-10, 2020. Online at: http://www.cs.wpi.edu/~claypool/papers/eeq/*

In addition, both the English original of the EEQ and the EEQ-G, as well as other translations, are available at https://web.cs.wpi.edu/~claypool/papers/eeq/

Version 1.0, 7 December 2022
DOI: https://doi.org/10.1371/journal.pone.0286556

The Exergame Enjoyment Questionnaire – German Version (EEQ-G)

Der EEQ-DE ist ein Fragebogen zur Erfassung der Freude am Spielen von Exergames, welcher unmittelbar nach dem Spielen von Exergames angewandt werden soll.

Bewertung

Der EEQ-G wird durch das Aufsummieren der Punkte für jede Frage bewertet.

Für die Fragen 1, 2, 3, 5, 7, 8, 12, 14, 15, 16, 17 und 20 werden die Punkte wie folgt vergeben:

Antwort:	Punkte:
Stimme überhaupt nicht zu	1
Stimme eher nicht zu	2
Neutral	3
Stimme eher zu	4
Stimme voll und ganz zu	5

Für die Fragen 4, 6, 9, 10, 11, 13, 18 und 19 werden die Punkte wie folgt vergeben:

Antwort:	Punkte:
Stimme überhaupt nicht zu	5
Stimme eher nicht zu	4
Neutral	3
Stimme eher zu	2
Stimme voll und ganz zu	1

Daraus resultierend ergibt sich eine minimale Punktzahl von 20 und eine maximale Punktzahl von 100. Eine höhere Punktzahl spiegelt eine grössere Freude am Spielen der Exergames wieder.

Referenz:

Der EEQ-G soll mit folgender Referenz zitiert werden:

Manser P, Huber S, Seinsche J, de Bruin E D, Giannouli E (2023) Development and Initial Validation of the German Version of the Exergame Enjoyment Questionnaire (EEQ-G). PLOS ONE. https://doi.org/10.1371/journal.pone.0286556

DOI: https://doi.org/10.1371/journal.pone.0286556

	The Exergame Enjoyment Questionnaire – German Version (EEQ-G)	Stimme überhaupt nicht zu	Stimme nicht zu	Neutral	Stimme zu	Stimme voll und ganz zu
1	Ich war begeistert von den körperlichen Aktivitäten während des Spielens.	○	○	○	○	○
2	Durch das Training mit den Exergames habe ich mich gut gefühlt.	○	○	○	○	○
3	Ich habe mich gefühlt als hätte ich die Zeit während des Spielens der Exergames vergessen.	○	○	○	○	○
4	Ich fand es schwierig zu verstehen, wie die Exergames funktionieren.	○	○	○	○	○
5	Ich war auf die Exergames fokussiert.	○	○	○	○	○
6	Ich fand, die Spiele hätten ohne körperliche Aktivität mehr Spass gemacht.	○	○	○	○	○
7	Für mich war es leicht, mich mit der Spielsteuerung vertraut zu machen	○	○	○	○	○
8	Ich fühlte mich emotional mit den Exergames verbunden.	○	○	○	○	○
9	Ich empfand das Spielen der Exergames als Training.	○	○	○	○	○
10	Ich empfand die körperliche Aktivität als zu intensiv für mich.	○	○	○	○	○
11	Ich verspürte keinen Wunsch, Fortschritte in den Exergames zu machen.	○	○	○	○	○
12	Ich hatte das starke Gefühl, in der Welt der Exergames zu sein, bis hin zu dem Punkt, an dem ich meine Umgebung nicht mehr bewusst wahrnahm.	○	○	○	○	○
13	Ich hätte lieber nicht trainiert, obwohl das Training von Spielelementen begleitet war.	○	○	○	○	○
14	Ich empfand das Spielen der Exergames als nützlich für mein körperliches Wohlbefinden.	○	○	○	○	○
15	Ich fand, dass die Exergames eine angenehme Herausforderung darstellten.	○	○	○	○	○
16	Ich hatte das Gefühl, durch das Spielen der Exergames etwas erreicht zu haben.	○	○	○	○	○
17	Ich fand, dass die Exergames schnell auf meine Handlungen reagierten.	○	○	○	○	○
18	Ich hatte keine Lust, die Exergames weiterzuspielen.	○	○	○	○	○
19	Ich hätte es bevorzugt, wenn die körperliche Aktivität nicht von Spielelementen begleitet gewesen wäre.	○	○	○	○	○
20	Ich hatte das Gefühl, die Kontrolle über die Exergames zu haben.	○	○	○	○	○

DOI: https://doi.org/10.1371/journal.pone.0286556

The Exergame Enjoyment Questionnaire (EEQ)

v1.0

The EEQ is a survey instrument to measure exergame enjoyment. Questions are intended to be administered to users immediately after playing an exergame.

Scoring

The EEQ is scored by adding points for each question.

For questions 1, 2, 3, 5, 7, 8, 12, 14, 15, 16, 17, 20 the points are as follows:

Answer	Points
Strongly Disagree	1
Disagree	2
Neutral	3
Agree	4
Strongly Agree	5

For questions 4, 6, 9, 10, 11, 13, 18, and 19 the points are as follows:

Answer	Points
Strongly Disagree	5
Disagree	4
Neutral	3
Agree	2
Strongly Agree	1

Thus, the minimum score is 20 points and the maximum score is 100 points. Higher is better, indicating more enjoyment.

Questions

1. I felt excited about the physical activities in the game.
   Strongly Disagree, *Disagree*, *Neutral*, *Agree*, *Strongly Agree*
2. The exercise in this game made me feel good.
   Strongly Disagree, *Disagree*, *Neutral*, *Agree*, *Strongly Agree*
3. I felt like I lost track of time while playing.
   Strongly Disagree, *Disagree*, *Neutral*, *Agree*, *Strongly Agree*
4. I felt that it was difficult to understand how the game works.
   Strongly Disagree, *Disagree*, *Neutral*, *Agree*, *Strongly Agree*
5. I was focused on the game.
   Strongly Disagree, *Disagree*, *Neutral*, *Agree*, *Strongly Agree*

DOI: https://doi.org/10.1371/journal.pone.0286556

6. I felt that the game would have been more enjoyable without physical activity.
   Strongly Disagree, *Disagree*, *Neutral*, *Agree*, *Strongly Agree*
7. I felt that it was easy to familiarize myself with the game controls.
   Strongly Disagree, *Disagree*, *Neutral*, *Agree*, *Strongly Agree*
8. I felt emotionally attached to the game.
   Strongly Disagree, *Disagree*, *Neutral*, *Agree*, *Strongly Agree*
9. I consider playing the game "exercise".
   Strongly Disagree, *Disagree*, *Neutral*, *Agree*, *Strongly Agree*
10. I felt that the physical activity was too intense for me.
    Strongly Disagree, *Disagree*, *Neutral*, *Agree*, *Strongly Agree*
11. I did not feel a desire to make progress in the game.
    Strongly Disagree, *Disagree*, *Neutral*, *Agree*, *Strongly Agree*
12. I felt a strong sense of being in the world of the game to the point that I was unaware of my surroundings.
    Strongly Disagree, *Disagree*, *Neutral*, *Agree*, *Strongly Agree*
13. I would rather not be exercising, even though the exercise was accompanied by game elements.
    Strongly Disagree, *Disagree*, *Neutral*, *Agree*, *Strongly Agree*
14. I felt that playing the game was beneficial for my physical well-being.
    Strongly Disagree, *Disagree*, *Neutral*, *Agree*, *Strongly Agree*
15. I felt that this game provided an enjoyable challenge.
    Strongly Disagree, *Disagree*, *Neutral*, *Agree*, *Strongly Agree*
16. I felt a sense of accomplishment from playing the game.
    Strongly Disagree, *Disagree*, *Neutral*, *Agree*, *Strongly Agree*
17. I felt that the game reacted quickly to my actions.
    Strongly Disagree, *Disagree*, *Neutral*, *Agree*, *Strongly Agree*
18. I did not feel like I wanted to keep playing.
    Strongly Disagree, *Disagree*, *Neutral*, *Agree*, *Strongly Agree*
19. I would prefer that this physical activity was not accompanied by game elements.
    Strongly Disagree, *Disagree*, *Neutral*, *Agree*, *Strongly Agree*
20. I felt in control of the game.
    Strongly Disagree, *Disagree*, *Neutral*, *Agree*, *Strongly Agree*

References

Alexander Fitzgerald, Sam Huang, Kyle Sposato, Dongjie Wang, Mark Claypool, and Emmanuel Agu. "The Exergame Enjoyment Questionnaire (EEQ): An Instrument for Measuring Exergame Enjoyment", In *Proceedings of the Hawaii International Conference on System Sciences (HICSS)*, Maui, Hawaii, January 7-10, 2020. Online at: http://www.cs.wpi.edu/~claypool/papers/eeq/

DOI: https://doi.org/10.1371/journal.pone.0286556
